# RNA sequencing reveals differentially expressed genes as potential diagnostic and prognostic indicators of gallbladder carcinoma

**DOI:** 10.18632/oncotarget.3861

**Published:** 2015-04-28

**Authors:** Xing Gu, Bin Li, Mingming Jiang, Meng Fang, Jun Ji, Aihua Wang, Mengmeng Wang, Xiaoqing Jiang, Chunfang Gao

**Affiliations:** ^1^ Department of Laboratory Medicine, Eastern Hepatobiliary Surgery Hospital, Second Military Medical University, Shanghai, 200438, PR China; ^2^ Department of Biliary Tract Surgery I, Eastern Hepatobiliary Surgery Hospital, Second Military Medical University, Shanghai, 200438, PR China

**Keywords:** biomarker, oncology, oncogene, tumor suppressor, tumorgenesis

## Abstract

Gallbladder carcinoma (GBC) is a rare tumor with a dismal survival rate overall. Hence, there is an urgent need for exploring more specific and sensitive biomarkers for the diagnosis and treatment of GBC. At first, amplified total RNAs from two paired GBC tumors and adjacent non-tumorous tissues (ANTTs) were subjected to RNA sequencing. 161 genes were identified differentially expressed between tumors and ANTTs. Functional enrichment analysis indicated that the up-regulated genes in tumor were primarily associated with signaling molecules and enzyme modulators, and mainly involved in cell cycles and pathways in cancer. Twelve differentially expressed genes (DEGs) were further confirmed in another independent cohort of 35 GBC patients. Expression levels of *BIRC5*, *TK1*, *TNNT1* and *MMP9* were found to be positively related to postoperative relapse. There was also a significant correlation between *BIRC5* expression and tumor-node-metastasis (TNM) stage. Besides, we observed a positive correlation between serum CA19–9 concentration and the expression levels of *TNNT1*, *MMP9* and *CLIC3*. Survival analysis revealed that GBC patients with high *TK1* and *MMP9* expression levels had worse prognosis. These identified DEGs might not only be promising biomarkers for GBC diagnosis and prognosis, but also expedite the discovery of novel therapeutic strategies.

## INTRODUCTION

Gallbladder carcinoma (GBC), as the fifth most common gastrointestinal cancer, is a relatively uncommon but challenging cancer. In these years its incidence is increasing in China [1]. The risk factors for GBC include gender, aging, obesity, chronic cholecystitis and cholelithiasis [1–3]. Besides, various single nucleotide polymorphisms (SNPs) have also been determined to be associated with the susceptibility to GBC [4, 5]. GBC is the most common cause of mortality among biliary tract cancers (BCTs). Because patients with GBC often diagnosed at an advanced stage for lack of effective early diagnostic method, prognosis of GBC is generally dismal. The median survival time for individuals with GBC is no more than one year [2]. In spite of multiple studies on novel molecules for diagnosis and prediction of clinical outcomes in GBC [6], the progress in clinical applications has so far been limited. Thus, more precise markers with better sensitivity and specificity for GBC are needed to benefit the patients and expedite the discovery of novel therapeutic strategies.

RNA sequencing (RNA-Seq) technology, as a typical kind of next-generation RNA sequencing, was widely used recently in the studies of oncology, taking advantage of its superior sensitivity, high efficiency and high throughput. Many promising findings have been reported on the underlying molecular etiology or therapeutic targets in diverse malignancies, including prostate cancer [7], liver cancer [8], lung cancer [9], etc. However, there are few reports on the application of RNA-Seq in GBC study.

Here we used RNA-Seq technology to sequence the whole transcriptomes of paired tumor tissues and adjacent non-timorous tissues (ANTTs) from two GBC patients. The differentially expressed genes (DEGs) were further confirmed in another cohort of 35 GBC patients. The association of several DEGs with clinical characteristics and postoperative outcoming were also revealed.

## RESULTS

### General information of RNA-Seq and DEGs analysis

**Figure 1:**
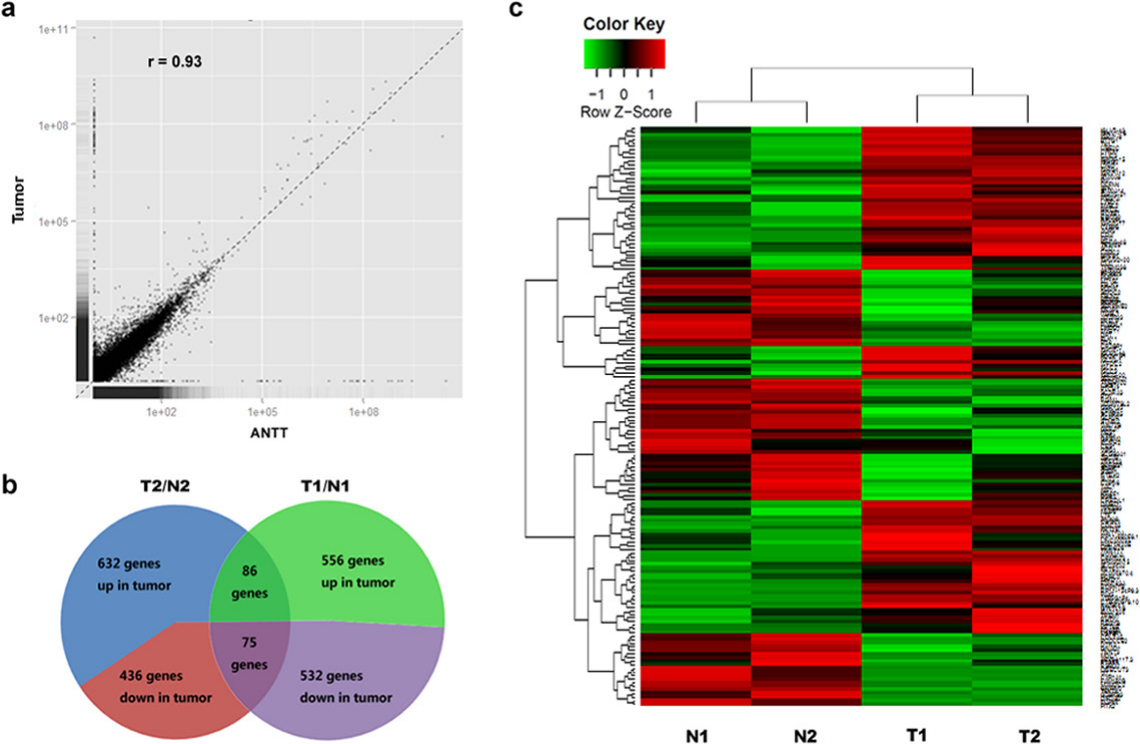
Schematic view of DEGs in GBC detected by RNA-Seq **a.** Expression analysis of GBC tumor and ANTT samples. The Pearson correlation coefficient is shown. **b.** Composition of DEGs in two paired samples used for RNA-Seq. **c.** Heat map was generated from normalized FPKM of 161 consistent DEGs across two paired samples. Expression level for each transcript is represented by a color range from green (low) to red (high).

RNA-Seq was performed on two pairs of matched GBC tumors and ANTTs, and the characteristics of the GBC patients were summarized in Table 2. There was no significant difference between numbers of genes detected in each pair of tumor and ANTT. An average of 16764 genes were detected in sequenced tissue sample, by requiring that the FPKM value was greater than 0.7. The correlation analysis showed that the average global profiles of gene expressions between tumor and ANTT samples are highly correlated (correlation coefficient *r* = 0.93; Figure 1a). Furthermore, the Q30 value of every library was more than 80%. The evaluations of sequencing randomness and saturation also proved the RNA-Seq system is good in quality.

**Table 1:** Demographic and laboratory parameters of the subjects included in the study

Samples	Validation Cohort	RNA-seq cohort	
	(*n* = 35)	T1/N1	T2/N2
Demographic parameters			
Gender (M/F)	15/20	M	F
Age (Y) ($\bar{x}$ ± SD)	59.66 ± 7.69	80	56
TNM stages		III	IV
II	2		
III	23		
IV	10		
Cholecystolithiasis	17	positive	positive
Laboratory parameters [median (range), Positive percentage]			
T-Bil (μmol/L)	12.4 (3.9–441.1), 10/35	6	227.6
D-Bil (μmol/L)	5.25 (1.4–341.1), 13/34	2.4	184.5
ALP (U/L)	86 (38–539), 13/35	99	1096
AFP (μg/L)	2.3 (0.7–187.9), 2/35	2.5	1.2
CEA (μg/L)	3.9 (0.6–91.3), 5/35	57.5	277.4
CA19-9 (U/ml)	157.3 (1.1– > 1000), 24/35	>1000	404.1
CA242 (IU/ml)	71 (0.8–200), 13/22	150	104.7

TNM stages, Tumor Node Metastasis stages; T-Bil, serum total bilirubin level; D-Bil, serum direct bilirubin level; ALP, serum alkaline phosphatase; AFP, serum alpha fetoprotein level; CEA, serum carcinoembryonic antigen level.

**Table 2:** The characteristics summary of the RNA-Seq data

Library	total reads	reads length(bp)	Total mapped	mapped%	mapped pairs	mapped paired%	Mapped genes^*^
T1	23,708,937	101	33,274,003	70.17%	15,623,535	65.90%	15,594
N1	16,433,264	101	18,224,025	55.45%	8,108,072	49.34%	16,245
T2	46,059,548	101	38,593,916	83.79%	18,494,595	80.31%	17,549
N2	40,830,844	101	34,288,810	83.98%	16,370,875	80.19%	17,584

* FPKM > 0.7 was defined as a mapped gene.

The expression levels of 632 and 556 genes were observed up-regulated in tumor tissues (T1/T2) compared with paired ANTTs (N1/N2) respectively. Meanwhile the expression levels of 436 and 532 genes were down-regulated (Figure 1b). Among all those genes, there were 86 up-regulated and 75 down-regulated consistently in both GBC pairs. Then these 161 DEGs were regarded as candidates for further study, whose expression levels were shown as heat map in Figure 1c.

### Functional enrichment analysis of DEGs

**Figure 2:**
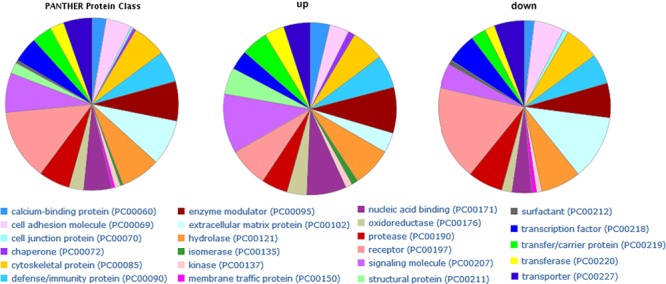
PANTHER protein class categories of total DEGs, increased and decreased DEGs in pie chart The chart legends showed each detailed category.

Functional enrichment analysis was used to investigate DEGs in GBC tumorigenesis. From GO results, 56 terms composed of 13 of cellular compound (CC), 12 of molecular function (MF), and 31 of biology process (BP) were significantly over-represented for all the DEGs (adjusted *P* values < 0.01) (Supplementary Table S2). For protein class by PANTHER website, the up-regulated genes in tumor tissues were mainly associated with “signaling molecule and enzyme modulator”, while the genes with decreased expression in tumor tissues were mainly associated with “receptor and extracellular matrix protein” (Figure 2). For KEGG pathway analysis, significantly enriched pathways of the up-regulated genes are “cell cycle” and “pathways in cancer”.

### Validation of RNA-Seq data by qRT-PCR

**Figure 3:**
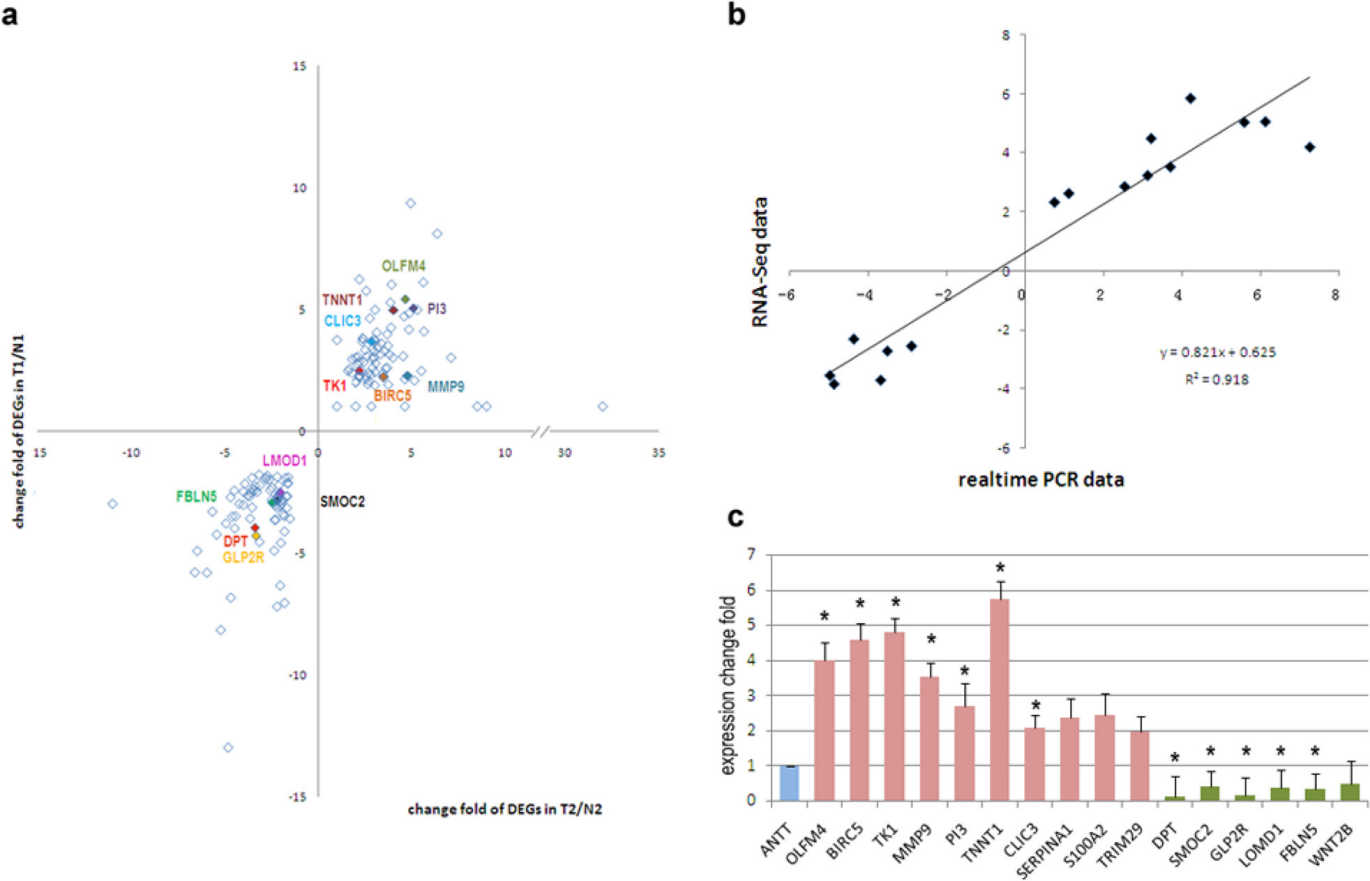
Validation of DEGs in other 35 paired tissue samples **a.** Validated DEGs by realtime PCR (colored) in all 161 DEGs. **b.** Correlation of expression of DEGs in RNA-Seq and validation cohort. **c.** Expression levels of DEGs in validation cohort, including up-regulated in tumor tissues (red) and down-regulated in tumor tissues (green). Expression level in ANTTs was used as a reference (blue). *, significant differences (*P* < 0.05).

To verify the RNA-Seq data, the original two RNA samples used for RNA-Seq were tested again by qRT-PCR on a panel of fifteen selected DEGs (Figure 3a). Log2 fold change of genes of qRT-PCR was compared with that of RNA-Seq. Finally, these DEGs were demonstrated concordant expression change in these two gene expression analysis platforms, with the correlation coefficient of validation cohort and RNA-Seq cohort was 0.958 (Figure 3b). Hence, our RNA-Seq method could reliably measure gene expression differences.

**Figure 4:**
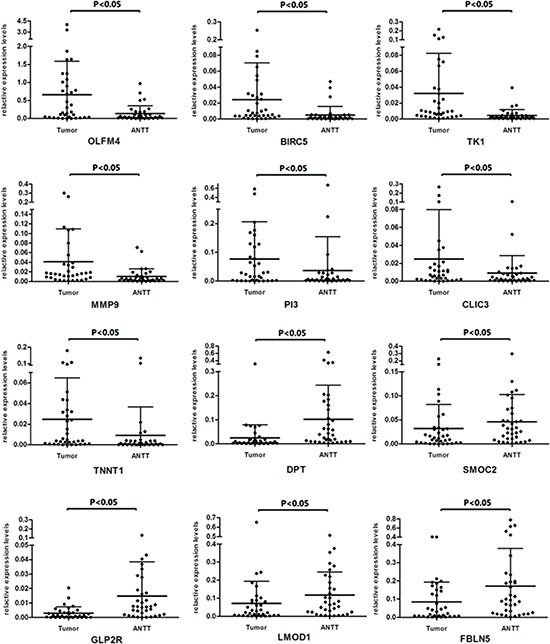
Expression levels of twelve DEGs in paired GBC tissues and ANTTs respectively

On the other hand, confirmation of RNA-Seq data was performed by qRT-PCR using samples from another independent cohort of 35 patients diagnosed as GBC. Among the selected 15 DEGs, 12 genes showed consistent expression differences in validation cohort as shown by RNA-Seq data (Figure 3c). Among the identified genes, *OLFM4*, *BIRC5*, *TK1*, *PI3*, *TNNT1*, *CLIC3* and *MMP9* levels were distinctly increased in GBC tissues. Meanwhile expression of gene *DPT*, *SMOC2*, *GLP2R*, *FBLN5* and *LMOD1* were down-regulated in tumor tissues. Figure 4 represented the expression levels of each validated DEG in paired GBC tissues and ANTTs respectively.

### Correlation analysis between DEGs and clinic pathological characteristics

Stratification analysis of DEGs up-regulated in tumor tissues was performed according to the GBC patients' preoperative laboratory and pathologic parameters. As shown in Table 3, the expression levels of *OLFM4* and *TK1* were both significantly positively related to patients' age. The expression levels of *BIRC5*, *TK1*, *TNNT1* and *MMP9* were found to be positively related to tumor recurrence after cystic resection. Besides, we observed a positive correlation with serum CA19–9 value and the expression levels of *TNNT1*, *MMP9* and *CLIC3*. There was also a positive correlation between another serum tumor marker CEA and *PI3* expression levels. Furthermore, *BIRC5* expression level was found to be correlated with tumor-node-metastasis (TNM) grade in GBC patients. The patients with higher TNM grades possessed more BIRC5 expression in tumors. However, the presence of cholelithiasis had no correlation with expression levels of these DEGs.

**Table 3:** Correlation analysis between DEGs up-expressed in GBC with clinical characteristics

Gene		Age	Gender	Cholelithiasis	TNM grade	relapse	Tbil	TBA	ALT	AST	GGT	ALP	CEA	CA199	CA242
OLFM4	Spearman Correlation	–0.435^**^	0.118	0.122	0.033	–0.011	0.106	0.125	0.039	0.015	0.027	0.038	0.035	0.165	–0.134
	Sig. (2-tailed)	**0.007**	0.488	0.474	0.845	0.966	0.534	0.495	0.819	0.931	0.875	0.823	0.837	0.329	0.506
BIRC5	Spearman Correlation	–0.260	0.118	–0.086	0.388^*^	0.494^*^	–0.051	–0.007	0.028	0.183	–0.037	0.034	0.096	0.160	0.076
	Sig. (2-tailed)	0.120	0.488	0.612	**0.018**	**0.037**	0.762	0.968	0.868	0.279	0.829	0.843	0.572	0.346	0.707
TK1	Spearman Correlation	–0.401^*^	0.194	–0.015	0.260	0.824^**^	–0.165	–0.226	–0.081	0.009	–0.151	–0.059	0.060	0.187	0.184
	Sig. (2-tailed)	**0.014**	0.250	0.929	0.120	**< 0.001**	0.329	0.213	0.636	0.958	0.374	0.730	0.724	0.267	0.358
MMP9	Spearman Correlation	0.170	–0.062	–0.086	0.142	0.653^**^	0.186	0.153	0.230	0.198	0.168	0.213	0.020	0.430^**^	0.349
	Sig. (2-tailed)	0.321	0.718	0.620	0.408	**0.004**	0.276	0.410	0.176	0.246	0.328	0.213	0.906	**0.009**	0.075
TNNT1	Spearman Correlation	–0.191	0.036	–0.046	0.072	0.626^**^	–0.052	–0.143	–0.125	–0.102	–0.245	–0.070	–0.025	0.494^**^	0.172
	Sig. (2-tailed)	0.258	0.834	0.789	0.672	**0.005**	0.761	0.434	0.459	0.550	0.144	0.680	0.884	**0.002**	0.391
PI3	Spearman Correlation	–0.254	0.312	–0.010	0.277	–0.011	–0.033	0.158	–0.029	0.026	–0.004	0.118	0.340^*^	0.167	–0.244
	Sig. (2-tailed)	0.129	0.060	0.953	0.097	0.966	0.846	0.388	0.863	0.879	0.980	0.487	**0.039**	0.322	0.219
CLIC3	Spearman Correlation	–0.241	0.110	0.121	0.255	0.477	0.208	–0.090	0.002	0.018	–0.002	0.131	0.102	0.505^**^	0.292
	Sig. (2-tailed)	0.158	0.522	0.484	0.133	0.053	0.224	0.630	0.992	0.919	0.989	0.448	0.555	**0.002**	0.148

* *P* < 0.05;

** *P* < 0.01.

### Survival analysis

**Figure 5:**
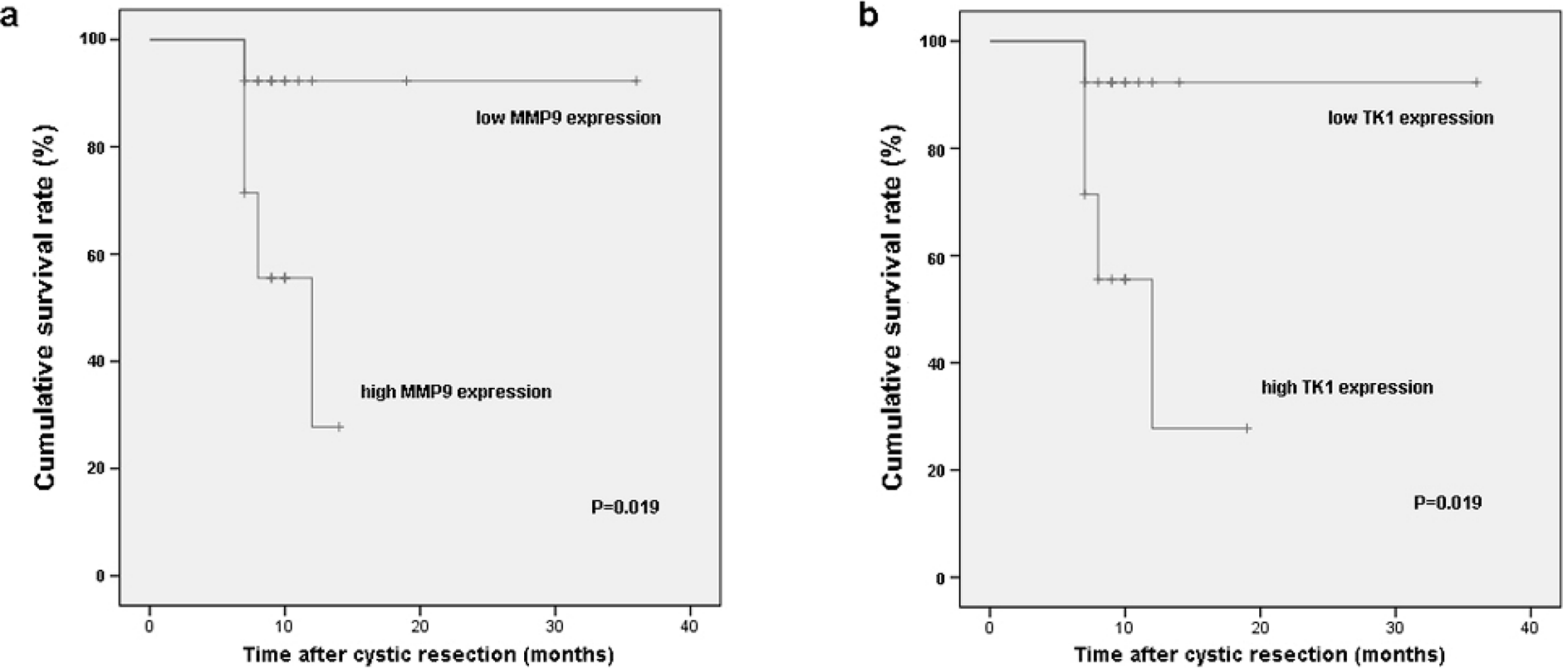
Survival rates of GBC patients who underwent cystic resection were compared between the high and low expression of *MMP9* a. and *TK1* b. The median expression level was used as the cutoff

The median follow-up duration in all involved 27 GBC patients was 10 months (range 7–36 months). Kaplan–Meier analysis revealed that survival rate of GBC patients with high *MMP9* expression was lower than that with low *MMP9* expression (*P* = 0.019) (Figure 5a). The expression level of *TK1* gene was found to bear similar effect on survival rate in present cohort. GBC patients with high *TK1* expression had worse prognosis (*P* = 0.019) (Figure 5b). However, there were no statistically significant differences between subgroups with different expression levels of the other DEGs.

## DISCUSSION

GBC is a highly malignant and fatal disease with a mean 5-year survival rate of 5% [10]. Its dismal prognosis attributes to its late presentation, early lymph node metastases, adjacent organ invasion, and poor response to chemotherapy. To improve the prognosis of GBC, it is important to screen out the risk factors or precursor diseases relevant to gallbladder carcinogenesis, and establish appropriate molecular biomarkers, favoring early diagnosis and progress monitoring.

Next generation sequencing (NGS) of the complete RNA transcriptome offers a novel approach for systematically characterizing the underlying molecular etiology of malignancies. Here by RNA-seq, we gained a whole transcriptomic profiling of GBC. To our knowledge, this is the first description of the global transcriptome of human gallbladder tumor tissue using RNA-seq approach. We observed hundreds of genes demonstrating 2-fold or greater increase or decrease in expression compared to matched ANTTs, respectively. Several candidate DEGs were screened out and further confirmed in another cohort of 35 GBC patients. Among these genes, some have already been investigated for their physiological function in gallbladder carcinogenesis, even tumorigenesis.

*BIRC5*, baculoviral IAP repeat containing 5, is a member of the inhibitor of apoptosis (IAP) gene family [11]. This gene encodes protein Survivin, which is famous for its crucial roles in apoptosis and cell cycle regulation [12]. Survivin over-expressed in a wide variety of tumors, accompanied by worse prognosis, shorter survival and increased tumor recurrence [13, 14]. Furthermore, Survivin is involved in the promotion of angiogenesis, which is necessary for tumor progression and expansion, by inhibiting apoptosis of endothelial cells. Recently, it has already been considered as a new diagnostic and prognostic marker of GBC [15]. As for *TK1*, thymidine kinase 1, its diagnostic and prognostic role of *TK1* has recently been widely investigated. *TK1* might be helpful for screening and monitoring of human malignancies [16, 17]. The increased expression of *TK1* in tumor is consistent with the former reports. Furthermore, an age-related difference was also observed in our study, which is in accord with Li's finding that more high serum *TK1* values were noted in the elderly patient population than low *TK1* levels [18]. Another familiar DEG in oncology is *MMP9*, matrix metallopeptidase 9, which promotes the progression of diverse cancers by improving tumor growth, migration, invasion, metastasis and angiogenesis [19, 20]. In tumor biology, it has been increasingly appreciated that *MMP9* is associated with poor disease prognosis, such as glioma [21] and gastric cancer [22]. *OLFM4*, olfactomedin 4, has been reported to be over-expressed in various cancers including gastric cancer but also colon, breast, and lung cancers [23], and has been proposed as a potential serum biomarker of gastric cancer [24]. Functionally, OLFM4 has been shown to bind to GRIM19 (a cell-death regulatory protein), cadherins and lectins, and could suppress apoptosis and promote tumor growth and invasion [23, 25]. However, a recent finding showed that *OLFM4* has a proapoptotic effect in myeloid leukemia cells [26]. Here, we found *OLFM4* as well as *TK1* was significantly positively related to patients' age. In previous report, positive expression of *OLFM4* was already found to be correlated with age in patients with gastric cancer [27], which is consistent with our result. Gene *PI3* is either called *ESI* (Elastase Specific Inhibitor) or *SKALP* (skin-derived antileukoprotease) or *Trappin-2* or *Elafin*, encoding an elastase-specific inhibitor. *PI3* is reported to participate in inflammatory responses e.g. by increasing neutrophil chemotaxis in situ while preventing the excessive protease damage generated by inflammation. This dual mechanism of action suggests that it acts at both ends of the inflammatory process [28]. As for its biological function in tumorigenesis, transcripts of *PI3* are extremely abundant in glioblastoma patient samples and significantly correlated with dismal survival [29]. In ovarian and breast carcinoma, *PI3* expression was also found to be transcriptionally up-regulated through activation of the NFκB and MEK/ERK signaling pathway, and correlate with unfavourable overall survival [30]. However, controversial report has already emerged that *PI3* could suppress tumorigenesis through inhibition of elastase and thus could serve as a prognostic indicator in breast cancer [31]. Chloride intracellular channel 3 (*CLIC3*) is a member of the p64 family and facilitates chloride ion movement and regulates cellular processes associated with the movement of chloride in placental and fetal membrane cells [32]. In addition, this protein may participate in cellular growth control, based on its association with ERK7, a member of the MAP kinase family [33]. *CLIC3* expression predicts lymph node metastasis and poor prognosis in operable cases of pancreatic ductal adenocarcinoma (PDAC) [34]. The finding indicated that *CLIC3* might be a potential molecule to promote cancer progression.

Compared with above increased genes detected in GBC, the role of gene *TNNT1* in carcinogenesis has been little unraveled. *TNNT1*, troponin T type 1, encodes a protein that is a subunit of troponin, which is a regulatory complex located on the thin filament of the sarcomere [35]. This complex regulates striated muscle contraction in response to fluctuations in intracellular calcium concentration. Serum *TNNT1* levels had a positive association with increased risk for hypertrophic cardiomyopathy [36] and also with different conditions related to the severity of the disease [37]. However, most studies on *TNNT1* are about its function in cardiovascular diseases [38, 39], whether it is involved in tumorigenesis is rarely known.

In this study, we also found correlations between these up-regulated DEGs and clinical characteristics by stratification analysis. In spite of some verified DEGs have already been studied intensively, most of their function and clinical application have not been previously reported in GBC. The correlation between *TNNT1* expression and tumor progression (TNM grade) or serum tumor marker CA19–9 might catch more attention on its potential application in diagnosis for GBC. The association between *BIRC5*, *TK1*, *TNNT1* and *MMP9* expression and postoperative relapse, together with the repression of *TK1* and *MMP9* expression on survival rate suggested their clinical prognostic value for GBC patients. However, only 27 GBC patients had intact follow-up information. The overall survival may have been influenced by the limitation of patients' number. Further follow-up studies with a large amount of GBC patients are needed to make up this shortcoming and confirm the effect of these DEGs expression on prognosis.

Although we put more attention on the increased DEGs, the verified decreased DEGs are also very noteworthy. The most significant decreased gene in cancer samples is *DPT*, encoding protein Dermatopontin, an extracellular matrix protein with possible functions in cell-matrix interactions assembly [40], cell adhesion [41], wound healing [42], and positive modification of the growth inhibition activity of TGF-β1 [43]. *DPT* was significantly down-regulated in HCC tissues compared with non-tumor [44]. Down-regulation of *DPT* is also observed in oral squamous cell carcinoma (OSCC)-derived cells, which suggested that it might inhibit tumor invasion and metastasis [45]. *DPT* together with other decreased genes, which have been less investigated, might be potential tumor suppressor and need to be further studied.

Lately, Li reported their work about whole-exome and targeted gene sequencing of gallbladder carcinoma [46]. They identified several genes with a significant frequency of non-silent mutations including *TP53*, *KRAS* and *ERBB3*, and the most extensively mutated pathway ErbB signaling. Here we also checked expression levels of above genes in our RNA-Seq data. However, besides *CDKN2A*, which is extremely over-expressed in tumor tissues, other genes mentioned by Li did not show significant difference on transcription level. This discrepancy indicates the different significance between researches on transcriptome and exome. RNA-Seq offers another view of transcriptomics to make up the shortcoming of whole-exome sequencing on DNA level.

In conclusion, we screened out twelve DEGs from GBC tissues by RNA-seq and confirmed them in 35 GBC patients. The up-regulated DEGs in this study may be promising molecular candidates for gallbladder cancer diagnosis or prognosis. All these DEGs in GBC revealed a pool of novel molecules which could be available for furthermore investigation of mechanism for GBC development and progression, and even for targeted therapy.

## MATERIALS AND METHODS

### Patients' information

A total of 37 GBC patients were recruited for RNA-Seq and validation in this study, including the specimens of one male GBC patient (T1/N1) and one female GBC patient (T2/N2) used for RNA-Seq. Tumor tissues and paired ANTTs were collected at the time of operation. Pathological diagnosis was made by two independent and expert pathologists. All of the tumor tissues were confirmed as primary adenosquamous carcinoma of gallbladder. Demographic, preoperative laboratory and pathologic data of all patients were collected from electronic medical records (EMR) and retrospectively reviewed. The whole cohort consists of 16 men and 21 women, with a mean age of 59. Among these GBC patients there were fourteen patients with gallstones. According to the seventh edition of the Union for International Cancer Control (UICC)and American Joint Committee on Cancer (AJCC) on Tumor Node Metastasis (TNM) classification, twenty-three and thirteen of these patients presented at tumor-node-metastasis (TNM) stage III and IV respectively. The demographic and laboratory parameters of all subjects included in the present study were listed in Table 1. Tumor recurrence and survival data were collected during the follow-up study. Informed consents of all patients were obtained in advance, and the data were analyzed anonymously. This study was approved by the Committee on Ethics of Biomedicine Research, Eastern Hepatobiliary Surgery Hospital, Second Military Medical University. All experiments were conducted in accordance with the official recommendations of the Chinese Community Guidelines.

### RNA library preparation and sequencing

Total RNAs of GBC tumor tissues and paired ANTTs from two GBC patients were isolated using Trizol reagent (Life Technologies, USA). Four libraries were constructed using Illumina standard kit according to the manufacturer's protocol. All sequencing was performed on Illumina Hiseq2000.

### Read mapping and screening of DEGs

Tophat v2.0.0 with default parameters in pair-end mode was used to map reads to reference genomes (Ensembl Human Genome v73). Pair-end reads with one-end drop in one chromosome and the other end drop in different chromosome had been discarded. As for reads could be mapped to multiple positions, we uniformly divided each multi-mapped read to all of positions it mapped to. In other words, a read mapping to 10 positions would count as 0.1 of a read at each position. Then, the expression levels for each gene normalized by fragments per kilo base of transcript per million mapped reads (FPKM) were calculated based on filtered mapping results. FPKM > 0.7 was defined as a mapped gene.

The numerical measure of mapped reads was used to evaluate the expression level of certain gene or alternative splicing isoforms using TopHat and Cufflinks. Expression difference of each gene between tumor tissues and ANTTs was analyzed based on Cufflinks. The significant DEGs were selected with threshold P-value less than 0.05 and fold-change more than 2 between each paired tumor and ANTT libraries. Meanwhile, their expression change tendencies were consistent in both two GBC patients for RNA-Seq. As the total number of reads varied between libraries, the fold-change was calculated after normalization.

### Functional enrichment analysis of DEGs

Functional enrichment analysis was used to investigate DEGs in GBC tumorigenesis. Gene ontology (GO) (http://www.geneontology.org/) is a standard classification system of gene function and gene products. Besides GO analysis, PANTHER website (http://go.pantherdb.org/) and Kyoto Encyclopedia of Genes and Genomes (KEGG) pathway analysis (http://www.kegg.jp/) were also used to reveal the physiological function of DEGs in gallbladder carcinogenesis.

### DEGs verification and stratification analysis

The differential expression genes (DEGs) were initially validated by quantitative real-time PCR (qRT-PCR) on the same set of RNA sequencing. Furthermore, total RNAs were extracted from tumor tissues and paired ANTTs of an independent cohort of 35 GBC patients using an RNeasy Mini Kit (Qiagen, the Netherlands). The concentration and quality of RNAs were measured using EpochTM (BioTek, USA). The cDNAs were synthesized using ReverTra Ace-α-RT-PCR kit (TOYOBO, Japan) according to the manufacturer's instructions. The qRT-PCR of DEGs was performed using the SYBR Green Real-time PCR Master Mix kit (TOYOBO, Japan) and was analyzed on ABI system 7300 (LifeTechnologies, USA). All assays were carried out independently in triplicate. The gene *ACTB* was used as the reference. Relative gene expression values expressed as fold change were subsequently determined using the Delta-Delta Ct method. The primers for qRT-PCR were listed in Supplementary Table S1.

Furthermore, the GBC patients were retrospectively stratified based on their clinical characteristics, including preoperative laboratory and pathologic parameters such as CA19–9, TNM stages and the presence of cholelithiasis. The presence of relapse was also included. Then, the expression levels of these validated DEGs were compared between subgroups to detect their relationship with clinical characteristics.

### Statistical analysis

All data were analyzed using SPSS 18.0 software. Data were presented as mean ± standard deviation (mean ± S.D.) or median and range as appropriate. The *t* test was used to compare data with normal distribution between two independent groups, while Mann-Whitney *U* test was used for non-normal data. Comparison between two-paired groups was carried out using paired *t* test or Wilcoxon signed ranks test when it is necessary. The correlation of the gene expression between GBC and ANTT samples was analyzed using Spearman's correlation, as well as the correlation between DEG expression levels and clinical characteristics. Survival curves were calculated using the Kaplan–Meier method and compared using the log-rank test. The chi-square test was used for categorical data. *P* < 0.05 was defined as indicating a statistical significance.

## SUPPLEMENTARY TABLES


