# Supplementary material for: RNA sequencing reveals differentially expressed genes as potential diagnostic and prognostic indicators of gallbladder carcinoma
Source: Oncotarget. 2015 Apr 28;6(24):20661–71. doi: 10.18632/oncotarget.3861 (PMC4653033; doi:10.18632/oncotarget.3861)
Supplement: Supplementary file 1 [file oncotarget-06-20661-s001.pdf]

SUPPLEMENTARY TABLES

Supplementary Table S1: Primer sequences for Realtime PCR

Gene ID	Gene	Upstream primer (5'-3')	Downstream primer (5'-3')	Size of product (bp)
ENSG00000105048	TNNT1	TGATCCCGCCAAAGATCCC	TCTTCCGCTGCTCGAAATGTA	123
ENSG00000112562	SMOC2	ATGACGACGGCACCTACAG	TCGCGTTGGGGTAACTTTTCA	154
ENSG00000163431	LMOD1	GTAAAAGGGGAGCGTAGGAAC	CTCGGGTGTTTTGGTCTTGCT	210
ENSG00000065325	GLP2R	TCCTGGAAATGTCTCTGTACCC	GGCGTTCTCTATCGTCTGCC	118
ENSG00000167900	TK1	GTTTTTCCCTGACATCGTGGA	CGAGCCTCTTGGTATAGGCG	200
ENSG00000102837	OLFM4	TCCCACTCCAGGGAGCTGTG	CATCTGTATTCAATGGCGCCAC	170
ENSG00000089685	BIRC5	TAAAAAGCATTCGTCCGGTTG	TTCTCCGCAGTTTCCTCAAAT	159
ENSG00000100985	MMP9	TCCAGTACCGAGAGAAAGCCT	TAGGTCACGTAGCCCACTTGG	103
ENSG00000124102	PI3	CGCTGGGACGCTGGTTCT	GCAGCGGTTAGGGGGATTC	235
ENSG00000169583	CLIC3	CCTCAAGGGCGTACCTTTCAC	GTCGCTGTCATAGAGCAGGA	112
ENSG00000137699	TRIM29	TGCGAGCTGCATCTCAAGC	GGTGCTATGATTCTTGTGCTCC	189
ENSG00000143196	DPT	TGACAGACAATGGAACTACGC	TGCTGTAGCGACAACAGTAAAA	203
ENSG00000140092	FBLN5	CTCACTGTTACCATTCTGGCTC	GACTGGCGATCCAGGTCAAAG	89
ENSG00000196754	S100A2	GCCAAGAGGGCGACAAGTT	GCTTCTTCAGCCCCTCCTCAT	110
ENSG00000134245	WNT2B	TACCTGCGGCGACGCTATG	ACAACCGTCTGTTCCTTTTGAT	216
ENSG00000075624	ACTB*	CATGTACGTTGCTATCCAGGC	CTCCTTAATGTCACGCACGAT	250

*The gene ACTB was used as the reference.

Supplementary Table S2: Gene ontology for functional enrichment analysis of DEGs

a. GO _BP

label	id	number_in_reference	number_in_list	expected	*p*Value
regulation of biological process	GO:0050789	9584	95	60.219	3.07E–07
biological regulation	GO:0065007	10036	97	63.059	6.80E–07
biological_process	GO:0008150	15838	125	99.514	1.01E–05
response to stimulus	GO:0050896	6965	74	43.763	1.37E–05
single-organism cellular process	GO:0044763	10610	97	66.665	2.15E–05
single-organism process	GO:0044699	11997	105	75.380	2.18E–05
multicellular organismal process	GO:0032501	5830	65	36.631	3.11E–05
positive regulation of biological process	GO:0048518	4029	50	25.315	9.12E–05
regulation of cellular process	GO:0050794	9097	85	57.159	2.32E–04
anatomical structure development	GO:0048856	4056	49	25.485	2.84E–04
humoral immune response	GO:0006959	128	8	0.804	3.17E–04
regulation of cell proliferation	GO:0042127	1302	24	8.181	3.57E–04
tissue development	GO:0009888	1317	24	8.275	4.34E–04
developmental process	GO:0032502	4643	53	29.173	4.87E–04
single-multicellular organism process	GO:0044707	5616	60	35.287	6.32E–04
regulation of cellular component organization	GO:0051128	1555	26	9.770	7.43E–04
single-organism developmental process	GO:0044767	4596	51	28.878	1.96E–03
multicellular organismal development	GO:0007275	4091	47	25.705	2.10E–03
response to lipid	GO:0033993	641	15	4.028	2.45E–03
positive regulation of cellular process	GO:0048522	3615	43	22.714	2.53E–03
system development	GO:0048731	3497	42	21.973	2.63E–03
extracellular matrix organization	GO:0030198	356	11	2.237	3.11E–03

(*Continued*)

a. GO _BP

label	id	number_in_reference	number_in_list	expected	*p*Value
extracellular structure organization	GO:0043062	357	11	2.243	3.19E–03
regulation of multicellular organismal process	GO:0051239	2010	29	12.629	3.22E–03
cellular response to stimulus	GO:0051716	5446	56	34.219	5.66E–03
regulation of cell growth	GO:0001558	318	10	1.998	6.54E–03
muscle contraction	GO:0006936	197	8	1.238	6.95E–03
negative regulation of biological process	GO:0048519	3522	41	22.130	7.28E–03
response to external stimulus	GO:0009605	1668	25	10.480	7.35E–03
cell differentiation	GO:0030154	2795	35	17.562	7.46E–03
cellular developmental process	GO:0048869	2917	36	18.328	7.62E–03

b. GO_MF

label	id	number_in_ reference	number_in_list	expected	*p*Value
glycosaminoglycan binding	GO:0005539	186	14	1.169	1.04E–09
heparin binding	GO:0008201	138	12	0.867	5.97E–09
sulfur compound binding	GO:1901681	201	12	1.263	3.90E–07
molecular_function	GO:0003674	15431	120	96.957	1.35E–04
protein binding	GO:0005515	8233	78	51.730	2.23E–04
enzyme inhibitor activity	GO:0004857	321	10	2.017	2.17E–03
peptidase regulator activity	GO:0061134	199	8	1.250	2.29E–03
extracellular matrix structural constituent	GO:0005201	66	5	0.415	3.68E–03
tropomyosin binding	GO:0005523	13	3	0.082	4.52E–03
peptidase inhibitor activity	GO:0030414	165	7	1.037	5.01E–03
receptor binding	GO:0005102	1253	20	7.873	6.36E–03
carbohydrate derivative binding	GO:0097367	2142	28	13.459	7.92E–03

(*Continued*)

c. GO_CC

label	id	number_in_reference	number_in_list	expected	*p*Value
extracellular region	GO:0005576	4061	73	25.516	6.11E–18
extracellular region part	GO:0044421	3331	63	20.930	1.01E–15
extracellular matrix	GO:0031012	393	23	2.469	4.59E–14
proteinaceous extracellular matrix	GO:0005578	327	21	2.055	1.54E–13
extracellular space	GO:0005615	1169	32	7.345	8.15E–11
cellular_component	GO:0005575	16877	134	106.042	3.70E–10
extracellular organelle	GO:0043230	2388	40	15.004	2.52E–07
extracellular vesicular exosome	GO:0070062	2388	40	15.004	2.52E–07
extracellular membrane-bounded organelle	GO:0065010	2388	40	15.004	2.52E–07
vesicle	GO:0031982	3142	43	19.742	1.99E–05
membrane-bounded vesicle	GO:0031988	3066	42	19.264	2.82E–05
interstitial matrix	GO:0005614	15	3	0.094	7.26E–03
extracellular matrix part	GO:0044420	124	6	0.779	8.39E–03
